# Cognitive rehabilitation in paediatric acquired brain injury—A 2-year follow-up of a randomised controlled trial

**DOI:** 10.3389/fneur.2023.1173480

**Published:** 2023-05-30

**Authors:** Hanna L. Sargénius, Stein Andersson, Ingvild Haugen, Ruth Hypher, Anne Elisabeth Brandt, Torun G. Finnanger, Torstein B. Rø, Kari Risnes, Jan Stubberud

**Affiliations:** ^1^Department of Psychology, University of Oslo, Oslo, Norway; ^2^Psychosomatic Medicine and Clinical Psychiatry, Oslo University Hospital, Oslo, Norway; ^3^Division of Mental Health Care, Innlandet Hospital Trust, Brumunddal, Norway; ^4^Department of Clinical Neurosciences for Children, Oslo University Hospital, Oslo, Norway; ^5^Department of Clinical and Molecular Medicine, NTNU, Trondheim, Norway; ^6^Children's Clinic, St. Olav University Hospital, Trondheim, Norway; ^7^Department of Research, Lovisenberg Diaconal Hospital, Oslo, Norway

**Keywords:** acquired brain injury, paediatric, goal management training, executive function, randomised controlled trial (RCT)

## Abstract

**Background:**

Goal management training (GMT), a metacognitive rehabilitation method that has been demonstrated to improve executive function (EF) in adults with acquired brain injury (ABI), could potentially be effective for children in the chronic phase of ABI. In a previously published randomised controlled trial (RCT), the efficacy of a paediatric adaptation of GMT (pGMT) compared to a psychoeducative control intervention (paediatric Brain Health Workshop, pBHW) was investigated. Comparable improvements in EF in both groups were found at 6-month follow-up. However, a specific effect of pGMT could not be conclusively proven. The present study reports 2-year follow-up data (T4; T1: baseline, T2: post-intervention, T3: 6-month follow-up, and T4: 2-year follow-up) from this original RCT.

**Methods:**

A total of 38 children and adolescents and also their parents completed questionnaires tapping into daily life EF. Explorative analyses were conducted comparing the 2-year follow-up data (T4) with the baseline (T1) and 6-month follow-up data (T3) for T4-participants in the two intervention groups (pGMT; *n* = 21, pBHW; *n* = 17), and we also assessed T4-participants vs. non-responders (*n* = 38) in the RCT. Primary outcome measures were the Behavioural Regulation Index (BRI) and the Metacognition Index (MI) derived from the Behaviour Rating Inventory of Executive Function (BRIEF) parent report.

**Results:**

No difference between intervention groups was found (BRI, *F* = 2.25, *p* = 0.143, MI, *F* = 1.6, *p* = 0.213), and no time^*^group interaction (BRI, *F* = 0.07, *p* = 0.976, MI, *F* = 0.137, *p* = 0.937) could be seen at the 2-year follow-up. Nevertheless, both pGMT and the pBHW groups improved daily EF as measured by parental reports over time from the baseline to T4 (*p* = 0.034). T4 participants and non-responders shared similar baseline characteristics.

**Conclusion:**

Our results extend the findings from the 6-month follow-up previously published. Both pGMT and pBHW groups sustained their improvements in daily life EFs from the baseline, but additional effectiveness of pGMT relative to pBHW was not found.

## 1. Introduction

Executive function (EF) is an umbrella term describing a complex set of cognitive processes necessary for an individual to control their behaviour. The neural systems underpinning EF are numerous, complex, and interrelated involving virtually all the brain regions in some way. As the infant's brain develops, the various components of EF progress in diverse developmental trajectories (1, 2). This development can be viewed as quantitative and qualitative changes in brain activity and brain organisation from infancy and into adulthood (3). Consequently, the young brain is especially vulnerable to any disruption or insult that can cause deviations from the typical developmental trajectory at this period of life (4, 5). Acquired cognitive and/or behavioural deficits following an insult may have a profound impact on the future of the child in various life domains (6, 7).

Acquired brain injury (ABI) is one of the leading causes of death and disability among children (8). ABI is a term that encompasses any damage to the brain that occurs after birth (9). This includes traumatic brain injury (TBI) and non-traumatic injuries such as brain tumours, cerebrovascular accidents, and infections. Whether it be TBI or non-traumatic insults, impairment to EFs is common across paediatric ABI (pABI) conditions. Children with pABI are found to experience problems with adaptive functioning and poorer quality of life (7, 10–14), experience executive dysfunction (15–17), and struggle with fatigue (12, 13, 18–23).

Cognitive difficulties, and, in particular, executive dysfunction, can have major consequences on a person's ability to live a functionally independent life. Compared to children with severe pABI, those with mild to moderate injuries can have cognitive problems that are too subtle to be detected in the initial stages following injury and thereby demonstrate relatively normal cognitive function (24). When problems are not detected and addressed properly, a consequence could be that the child is not able to adapt to or acquire the mental skills necessary to manage the increasing cognitive and social demands, leaving the children to fall behind their peers (25). Independent of the severity of the injury, children with pABI are at greater risk for unfavourable outcomes given the fact that there are currently no well-validated or standardised interventions for children with pABI and cognitive impairment, particularly after the initial critical care (26). Tailoring an optimal intervention for the child in question as early as possible is considered important as such actions could, for some children, ameliorate or even prevent executive dysfunction in the long term (9). In fact, the presence of persistent executive dysfunction could intensify the problems as time passes by (27), for instance, through behavioural dysregulation which will affect the child's quality of life and opportunities to participate in the community (28). Hence, there is undeniably a need to determine the long-term effectiveness of cognitive rehabilitation aimed to remediate EF difficulties.

Components such as metacognition and/or strategy use, drill-based exercises, and external aids/cuing appear to be the most used elements in cognitive rehabilitation of EF, either alone or combined within different types of interventions targeted towards children and adolescents (29–31). Interventions focusing on improving specific aspects of EF like attention and working memory and/or behavioural regulation are found to be relatively effective (32, 33). The involvement of caregivers and school services has been demonstrated to be central to the effectiveness of these interventions, especially for younger children (34). After all, those are the people who structure the child's daily activities and learning environment. Moreover, caring for a child with ABI will have a great impact on the family itself but also family functioning will affect potential recovery (35–37).

Goal management training (GMT) is a metacognitive group-based strategy training targeting attentional control and problem-solving in everyday situations (38). Emerging evidence has shown that GMT can improve EF when administered alone or in combination with supplementary interventions in adult populations with various neurologic and psychiatric conditions (39, 40). An advantage of GMT appears to be how improvements of EF are being expressed in terms of enhanced daily life function, such as perceived EF in everyday activity and social relations. This intervention also seems to positively influence various aspects of quality of life (41). Of late, GMT has come to attention due to its potential applicability for the cognitive rehabilitation of children with pABI. Although using GMT has been found feasible in some pABI studies (41, 42), these studies have been limited by small samples. Moreover, the complexity of GMT with its respective modules and approach requires metacognitive skills that might not be within reach for children at a young age and may constitute a challenge in direct applicability in paediatric conditions, and more research is warranted to determine the effectiveness and generalisation in this context (42).

The present study will provide 2-year follow-up data from a multicenter randomised controlled trial (RCT) (21, 43, 44). The objective of the original RCT was to investigate the effectiveness of GMT adapted for a paediatric population (pGMT) relative to an active comparison control condition, and the primary outcome was parent-reported daily life EF as measured with the Behaviour Rating Inventory of Executive Function (BRIEF) at the 6-month follow-up. In line with the original RCT, our explorative study will operate with the same outcome measure when investigating the long-term outcomes of the intervention in this cohort.

## 2. Methods

### 2.1. Participants and procedure

This study was a 2-year follow-up, and data were collected throughout August 2020. We invited all from the pre-registered national multicenter RCT conducted in 2018 (21, 43). A letter of information and questionnaires that have been administered at previous time points were distributed by regular mail to each child/teen–parent dyad. No diagnostic reassessment was done prior to participation in the 2-year follow-up.

A detailed description of the total sample in the original RCT, recruitment process, inclusion and exclusion criteria, classification of ABI, and study procedure has been provided in previous publications (43, 44). In brief, children in the chronic phase of pABI with EF complaints were recruited and assigned to either a metacognitive strategy training using pGMT (*n* = 38) or an active comparison control condition providing a psychoeducation paediatric Brain Health Workshop (pBHW, *n* = 38). The randomisation was in a 1:1 ratio, with block randomisation and stratification by (1) research site and (2) age at the time of intervention (10–13 years or 14–17 years). Blinding was applied to reduce systematic bias due to understanding of the treatment allocations, e.g., families and participants were not informed about the type of intervention, test technicians were blinded to treatment allocation, and therapists were blinded to all test performance. The investigators blinded to the intervention performed assessments at the baseline (T1), post-intervention (T2), and at 6-month follow-up (T3). The blinding of treatment allocation for the participants was still active at the 2-year follow-up analysis (T4).

Adherence was almost total at both T1 and T2 (73 out of 76 participants completed the baseline assessment and the allocated intervention). At the 6-month follow-up (T3), two participants (one participant in each of the pGMT and pBHW groups) failed to attend due to medical reasons. Leading up to the 2-year follow-up (T4), one participant had actively withdrawn from the study, and three participants had to withdraw from further participation due to a worsening of their medical condition, leaving 67 participants in total to be approached. Out of the 67 remaining participants, we received responses from *N* = 39 (58.2 %); one response was excluded due to missing subject ID, leaving in total 38 subjects (pGMT; *n* = 21, pBHW; *n* = 17) for analysis.

Injury aetiology, cognitive function, and psychosocial characteristics for the T4-sample are descriptively presented in Table 1. The mean age of the sample at T4 was 15.47 (*SD* = 2.48) years, the dominant causes of injury were cardiovascular accidents (*n* = 13), followed by brain tumour (*n* = 10), and TBI (*n* = 8). The mean age at onset was 7.71 (*SD* = 3.96) years, and the mean time since the injury was 5.32 years (*SD* = 2.73).

**Table 1:** T4-sample descriptives.

	**pGMT**	**pBHW**	**Total (*n* = 38)**
***Characteristics***			
Sex, boys/girls	No. 8/13	8/9	16/22
**Education level (mother)**			
<Primary school	1	0	1
High school	5	5	10
University level	11	9	20
>University, Master's degree	3	3	6
Age, *M*(*SD*)	15.33 (2.22)	15.64 (2.39)	15.47 (2.48)
Age at intervention, *M*(SD)	13.33 (2.22)	13.65 (2.4)	13.47 (2.27)
10–13 years (*n*)	12	8	20
14–17 years (*n*)	9	9	18
***Injury aetiology***			
Age at injury *M*(*SD*) years	7.90 (4.32)	7.47 (3.64)	7.71 (3.96)
Time since injury *M*(SD) years	4.95 (3.14)	5.76 (2.13)	5.32 (2.73)
**Primary injury (No.)**			
Traumatic brain injury	5	3	8
Brain tumour	5	5	10
Cerebrovascular accidents	8	5	13
Infection/inflammation	1	3	4
Hypoxia/Anoxia	2	1	3
***Intellectual abilities M (** ***SD*** **)***			
VCI	97.10 (12.85)	99.69 (14.55)	98.25 (13.49)
WMI	98.8 (10.3)	92.81 (14.48)	96.14 (12.51)
PSI	90.4 (15.35)	95.44 (16.03)	92.64 (15.64)
FSIQ	94.15 (13.06)	95.14 (14.41)	94.94 (13.51)
***Fatigue***			
PedsQL-MFS at baseline	57.36 (20.72)	57.9 (17.62)	57.6 (19.4)
PedsQL-MFS at T4	62.71 (19.86)	57.81 (23.1)	59.84 (21.25)

Intellectual abilities measured with WISC-V. Scores obtained for *n* = 37. VCI: Verbal Comprehension Index, WMI: Working Memory Index, PSI: Processing Speed Index, FSIQ: Full scale IQ. PedsQL-MFS Total score at baseline (pGMT, *n* = 20, pBHW, *n* = 16), PedsQL-MFS Total score at T4 (*n* = 38).

### 2.2. Interventions

**Figure 1:**
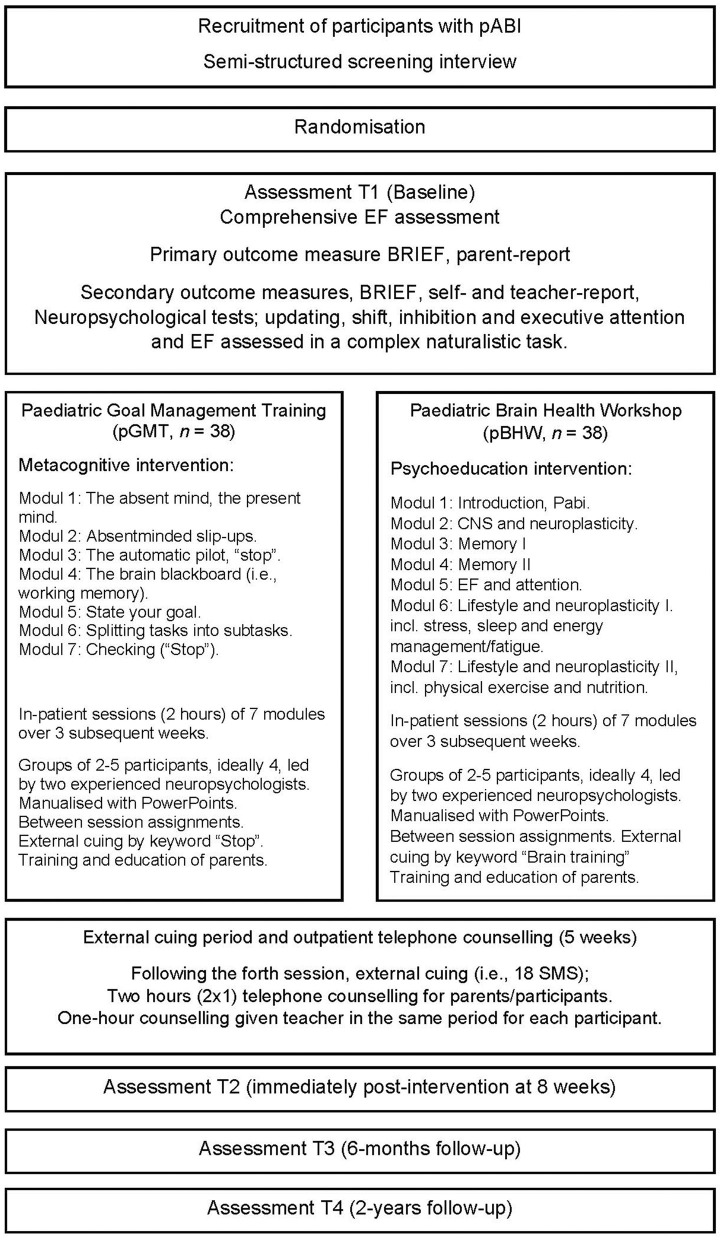
Outline of the study design. pABI, paediatric acquired brain injury; EF, executive function; BRIEF, Behaviour Rating Inventory of Executive Function; pGMT, paediatric Goal Management Training; pBHW, paediatric Brain Health Workshop.

The pGMT protocol used for the original RCT study was developed and piloted by Stubberud et al. (41) and put into action by Brandt et al. (43). Both the pGMT and pBHW interventions consisted of seven modules of 2 h duration led by experienced clinical neuropsychologists. Standardised PowerPoint presentations and workbooks were used, accompanied by both in-session practice and between-sessions exercises. The modules had to be completed in consecutive order (see Figure 1). Following the fourth session, the children received additional external cuing by text messages. Participants in both interventions were further divided into smaller groups of four children in each group. The groups provided the participants with a safe space for discussions concerning their own injuries and personal life experiences. The parents were offered group counselling and support throughout the process of applying the various techniques to everyday activities and asked to consecutively review the content of the intervention. For a more detailed description of the pGMT and pBHW modules, see the previously published study protocol (44).

### 2.3. Measure

#### 2.3.1. Descriptor measures

The baseline assessment of intellectual abilities was measured by the Wechsler Intelligence Scale for Children–Fifth Edition, WISC-V (45). In the present study, the Verbal Comprehension Index, the Working Memory Index, the Processing Speed Index, and the Full Scaled-IQ were calculated. Supplementary measures included (i) the Paediatric Quality of Life Inventory (PedsQL) (46), (parent- and self-report), a 23-item rating scale assessing physical, social, educational, and emotional functioning, each with a designated index, in addition to a total index score, (ii) the Paediatric Quality of Life Inventory–Multidimensional Fatigue Scale (PedsQL MFS) (parent- and self-reports), an 18-item questionnaire that describes symptoms of fatigue through three subscales (1) general fatigue, (2) sleep/rest fatigue, and (3) cognitive fatigue. Both questionnaires apply items that are rated for how frequently it is a problem on a 5-point scale from 0 “almost never” to 4 “almost always”. Scores ≤70 were defined as being in the clinical range (47).

#### 2.3.2. Primary and secondary outcomes

To assess daily life EF, the parents were asked to complete the BRIEF (48). To capture information on executive attention, an aspect of EF that is arguably not fully covered by the BRIEF, the ADHD Rating Scale IV (49) was additionally administered. The children/teens were given the equivalent questionnaire adaptations except for the ADHD-RS-IV (the self-reports did only function as supplementary and will not be discussed in this study). The children and their parents were all familiar with the various questionnaires used as they have been administered to them multiple times previously. Additionally, training in how to complete them has been provided.

The BRIEF parent report is a standardised rating scale assessing children's everyday EF in their natural home and school environments as perceived by their parents. It comprises 86 items, and each item is rated on a 3-point Likert scale from 1 (never) to 3 (often), with higher scores indicating greater dysfunctions. The Behavioural Regulation Index (BRI) and the Metacognition Index derived from BRIEF will be our primary outcome measures.

Supporting the BRI and MI, the Inattention Index from the ADHD-RS-IV is used as a secondary outcome measure. This index consists of nine questionnaire items related to situations and everyday activities which can be problematic for the child to carry out. Each item is rated on a 4-point Likert scale from 0 (never/rarely) to 3 (very often), with higher scores indicating greater attentional problems. The ADHD-RS-IV questionnaire itself comprises of in total 18-items and scores are summed up to score the total scale and the subscales, including the Inattention Index, and provides information on impulsivity and inattention (48).

### 2.4. Statistical analysis

SPSS 29.0 (IBM Corporation, Armonk, NY, USA) and JMP Pro 16.1.0 were used in all statistical analyses. The Mann–Whitney *U*-test and the chi-squares test were computed to detect potential differences in sociodemographic characteristics, intellectual abilities, and psychosocial functioning between the participants who completed T4 assessments and the non-responders. The T4 sample was compared to the non-responders relative to baseline data on the outcome measures and changes on the BRIEF parent report from T1 to T3.

For informative purposes only, an examination of the relationships between the BRIEF parent reports and BRIEF self-reports was conducted by computing a series of Spearman correlations using raw scores for the BRI and MI. After visual inspection of Q-Q plots of normality, the Mann–Whitney U-test was conducted to determine the discrepancy between parent reports and self-reports.

In the main analysis, the within and between-group differences of the primary outcome measures for the T4-sample were calculated by linear mixed modelling (LMM), using ARH(1) and restricted maximum likelihood (REML) for estimation. This model included the intervention group, time [baseline, post-intervention (T2), 6 months follow-up (T3), and 2-year follow-up (T4)], and the interaction of time and intervention as fixed factors. Due to the blinding and randomisation of participants to intervention groups, the assumption is that there is no systematic difference at the baseline. However, individual intercepts were included in the model as random effects to adjust for baseline values of the outcome variables. The statistical significance level was set to a *p*-value of <0.05. Because this study is explorative, Bonferroni corrections for multiple comparisons were only applied for the fixed factors in the LMM individually. Gender and fatigue total scores (parent report) with the clinical cut-off <70 were one at a time added as covariates in the analysis to adjust for potential influences on significant group^*^time interactions. However, neither gender nor fatigue did have any effect in the mixed model; thus, it was removed and left out from further analysis.

**Figure 2:**
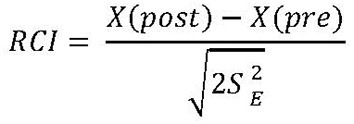
Standard error (*S*_*E*_) is calculated by using SD and coefficient alpha from the normative sample.

To gain insight into individual clinical change, a Reliable Change Index (RCI) was calculated for the primary outcome measures of the BRIEF parent report. The RCIs were calculated according to the formula in Figure 2, using each participant's pre-test and post-test scores and the standard deviations (*SD*) and coefficient alpha (*r*_nn_) from the normative sample for the scale (50).

#### 2.4.1. Bias

As in most research studies, we faced the problem of missing data, which may introduce biassed results as well as a loss of statistical power and precision to our study (51). In sections where it was possible to deduce from the structure of the questionnaire what some of the missing values should be (e.g., when the answer was put in between two options, the response representing the least pathology was chosen), it was possible to fill in most of the missing values (51). For the other scales, if data were available for at least 50% of the items on a scale, the mean was calculated for the available items. This corresponds to imputing the missing values by the average available items on a scale.

Based on the principle of intention-to-treat (ITT) and the assumption of missing data to be missing at random, available data for all 76 participants from the original RCT were entered into a separate LMM analysis (52). To ascertain the robustness of our primary model (T4 sample), *post hoc* sensitivity analyses were conducted in which multiple imputations (MI) were applied (53). The additional model for the total sample applied the same criteria for fixed and random effects as to the primary mixed model and was performed using both no imputation of data and 50 times repeated imputations performed before the mixed modelling procedure was conducted.

## 3. Results

### 3.1. Initial and descriptive analysis

The T4-sample and the non-responder group did not differ significantly on sociodemographic characteristics, injury etiology, IQ measures, or baseline scores for overall psychosocial functioning and fatigue symptoms (see Table 2). The two groups did not differ significantly on the BRIEF-BRI parent report at the baseline either, but a significant difference did occur for the BRIEF-MI although this was not the case when we adjusted for age and gender. Relative to score change for the primary outcome measures, no significant differences from the baseline to the 6-month follow-up (T3) between the two groups were found [For a detailed description of the total sample in the original RCT at baseline and T3, see the previous publication (43)].

**Table 2:** Characteristics of the T4-sample vs. non-responders of the 2-year follow-up.

	**T4-sample**	**Non-responders**	
**Baseline**	***M*** **(** ***SD*** **)**	***M*** **(** ***SD*** **)**	***P***
Age	13.47 (2.28)	13.29 (2.38)	0.726
FSIQ	94.94 (13.51)	90.03 (12.77)	0.132
Inattention index	9.99 (5.08)	11.27 (6.03)	0.369
PedsQL total	70.5 (15.64)	64.25 (18.16)	0.138
PedsQL-MFS total	57.6 (19.14)	53.34 (19.59)	0.504
BRIEF-BRI	43.45 (9.7)	48.11 (12.96)	0.112
BRIEF-MI	82.53 (13.71)	90.95 (18.91)	0.049^*^
**Change from T1 to T3**			
BRIEF-BRI	3.2 (7.64)	6.25 (6.68)	0.069
BRIEF-MI	5.97 (15.4)	8.21 (12.68)	0.503
Inattention index	2.03 (4.37)	2.0 (5.35)	0.974

All scores were reported as raw scores, except for the FSIQ scaled score. Change scores are calculated by subtracting 6-month follow-up scores from the baseline scores. All measures are derived from parent reports. The statistical significance level is at a *p-*value of < 0.05. ^*^Significant at 0.05.

### 3.2. Main analysis results

Both pGMT and pBHW groups show improved daily EF as measured by parental reports over time (BRI, *F* = 6.3, *p* = 0.001, MI, *F* = 3.49, *p* = 0.024). No main effect of group affiliation was found (BRI, *F* = 2.25, *p* = 0.143, MI, *F* = 1.6, *p* = 0.213), and there is no time^*^group interaction (BRI, *F* = 0.07, *p* = 976, MI, *F* = 0.137, *p* = 0.937 (Table 3). Furthermore, there appear to be unmeasured explanatory variables for each subject that raise or lower their performance in a way that appears random (Wald Z = 3.62, *p* < 0.001). This demonstrates the need for individual random intercepts in the model. The BRIEF self-reports comparing pGMT and pBHW revealed no difference at T4 mixed modelling which supports the primary results showing no differences between interventions (see Supplementary Table). The BRIEF self-report children's version was applied for all participants at T4 even though some of the adolescents had turned 18 of age (*n* = 8).

**Table 3:** Two-year outcome for the T4-sample.

**Measure**		**Baseline**	**8-week**	**6-month**	**24-month**	**Group**	**Time**	Group ^*^time
***Mean*** **[95%CI]**		**T1**	**T2**	**T3**	**T4**	***p***	***p***	***p***
BRIEF-BRI	GMT	45.33 [41.18–89.47]	43.79 [39.8–47.78]	42.0 [37.98–46.01]	40.52 [36.80–44.25]	0.143	0.001	0.976
	BHW	41.12 [36.50–45.73]	40.35 [35.96–44.75]	37.77 [33.3–42-23]	36.7 [32.57–40.84]			
	Total	43.22 [40.12–46.33]	42.07 [39.1–45.04]	39.88 [36.88–42.89]	38.61 [35.83–41.39]			
BRIEF-MI	GMT	84.9 [78.58–91.23]	81.19 [75.01–87.38]	77.95 [70.15–85.76]	78.33 [70.75–85.88]	0.213	0.024	0.937
	BHW	79.59 [72.56–86.62]	77.06 [70.25–83.87]	73.12 [64.44–81.79]	71.15 [62.75–79.54]			
	Total	82.25 [77.52–86.97]	79.13 [74.53–83.73]	75.53 [69.7–81.37]	74.74 [69.1–80.38]			
ADHD-RS-IV	GMT	10.52 [8.29–12.76]	N/A	8.76 [6.11–11.41]	9.33 [6.59–12.07]	0.207	0.02	0.818
Inattention	BHW	9.19 [6.63–11.6]	N/A	6.5 [3.59–9.48]	6.78 [3.68–9.88]			
	Total	9.82 [8.15–11.49]	N/A	7.63 [5.63–9.62]	8.06 [5.99–10.13]			

Fixed effects estimates, and estimated means with 95% CI for pGMT, pBHW, and total T4 sample. ADHD-RS-IV Inattention Index was not collected at T2 (post-intervention at 8-weeks). ^*^Significant.

**Figure 3:**
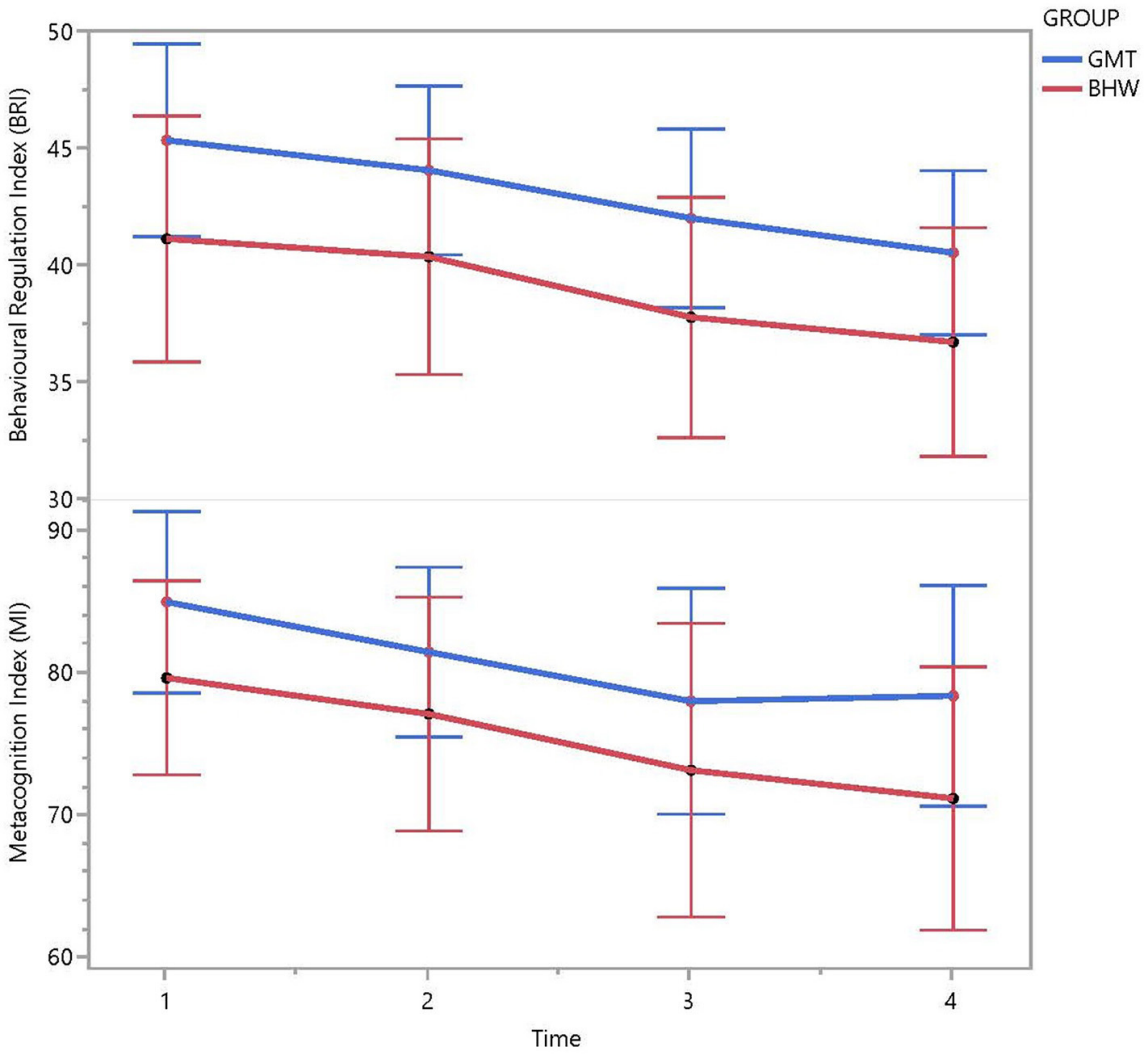
Behavioural Regulation Index (BRI) and Metacognition Index (MI) throughout T1-T4. Mean scores and 95% CIs reported for the pGMT and pBHW groups. Lower scores on the indexes indicate better cognitive function.

Concerning the Inattention Index for the overall T4-sample, a significant reduction from baseline to 6-month (*p* < 0.016), but not from 6-month to 2-year follow-up could be found. The relative improvement from T1–T4 as expressed by the Inattention Index, cannot be attributed to the type of intervention (*F* = 0.201, *p* < 0.818). Similar trends in how the Inattention Index changes over time, with an overall decrease from T1 to T3 in the estimated group means and a slight increase from T3 to T4 could be seen for both the pGMT and the pBHW groups. In sum, there seems to be no significant effect of the pGMT intervention compared to the pBHW (Figure 3).

Within-group changes over time on the primary outcome measures as expressed by RCI revealed similar trends for both the pGMT and pBHW groups, with some minor variations. A reliable clinical change for both the BRI and MI can be observed in eight pGMT participants and five pBHW group participants. However, among those from the pGMT group, two of the subjects had a negative change in the MI, and one had a negative change in both the BRI and MI. None of the participants from the pGMT had a reliable clinical change in the BRI only in contrast to the pBHW group in which three of the subjects had a reliable clinical change. One of these three subjects had a negative change. Four subjects from the pGMT (1 negative) and five subjects from the pBHW (2 negative) had a clinical change for the MI only.

## 4. Discussion

The objective of this exploratory study was to investigate whether pGMT could be more effective in terms of improving EF in daily life relative to a general psychoeducative treatment 2 years post-intervention as reported by parents in a pABI population. This is to find an intervention that is well-validated and standardised and that can be used for rehabilitation following pABI. In the original RCT, an overall improvement among the participants from both the pGMT and pBHW conditions for the primary outcome could be seen (changes in parent-reported BRIEF raw scores from baseline to 6-month follow-up). In line with these results, there are the results from the 2-year follow-up study with an overall trend of a sustained improvement of EF. This was the case for both the BRI and MI of the BRIEF parent report. Thus, pGMT did not demonstrate additional effectiveness on parent-reported daily life EF when compared to pBHW. Nevertheless, the fact that our study finds support for a sustained improvement in EF at 2 years post-intervention is appealing, considering that measures of functional outcome have been applied. Very few studies have investigated participant follow-up to establish if there is a maintenance of treatment effect from cognitive rehabilitation in pABI (28–30, 32). Moreover, to the best of our knowledge, this study is one of its kind in terms of being an RCT with a follow-up of the participants beyond the usual 6 or 12-month time points.

Studies prior to ours have demonstrated that independent of injury severity, children with a TBI will not fully recover to their preinjury level of functioning, and for certain subgroups, secondary acceleration worsens between 24 and 36 months for specific EF subscales (2, 54). Some EF subscales appear to worsen until 24 months and then plateau, and some subscales seem to improve after 24 months. In childhood brain tumour survivors, an observed decline in cognitive function as early as the first year following diagnosis and treatment is common, and for many, it is followed by a progression in cognitive problems over the next 5 to 7 years (5). These characteristic cognitive growth curves that seemingly are in play, provide us to some degree a comparison, allowing us to suggest that our interventions have been beneficial for our participants considering they have not regressed to a baseline functioning level.

It is not possible to isolate how our interventions may have contributed to this overall sustained improvement in our participants or why the participants in both intervention groups demonstrate the same tendency in maintenance. We propose, however, that it is the shared common factors rather than the differences between the two interventions which have resulted in the long-term effects that we have observed. A great effort was made to engage the children as much as possible, and both intervention groups had a combination of in-session practice and between-sessions exercises, and they all received outpatient telephone counselling in addition to external cueing. Adaptations of both pGMT and pBHW protocols were made to make the exercises and the phrasing used more accessible to the children and adolescents. Although developmental factors such as maturation and readiness and the motivation of the participants must be taken into consideration when verifying an intervention effect, as such factors will influence the degree to which the techniques and strategies learned will be internalised, we argue that by involving the family throughout the intervention, they have functioned as valuable scaffolders to the participating children and adolescent. Furthermore, in spite of the school only having a minor participating role in the intervention, their involvement may have been important for increasing awareness of pABI in their local community. Hence, we venture to suggest that the pGMT and the pBHW have initiated beneficial processes in the participants that are now being expressed way beyond the time of the intervention.

There may be several (interrelated) reasons why pGMT did not demonstrate a greater reduction in executive dysfunction in the present study. For one, the development of the young brain itself (according to chronological age and considering the time of injury) imposes a great challenge to the content and structure of an intervention and how it should be provided. For instance, in younger children, interventions most likely will benefit from being targeted towards core EFs skills rather than more complex higher-order skills that are yet to come (1, 55). Second, determining the effectiveness of a cognitive rehabilitation intervention is challenging given the chronic state of pABI. In contrast to the adult population, for children, skills that are yet to develop at the time of injury may not show impairments until years later, calling for long-term follow-up (55).

Another reason why pGMT did not outperform pBHW may be the potential effect of parent/child perception and expectations. It is well recognised that children with pABI have difficulties engaging in self-reflective processes and tend to overestimate their own abilities and are not aware of their actual level of functioning (56, 57). It is possible that the inherent difficulties in self-reflection that affect children with pABI limit the benefit of pGMT as this treatment requires metacognitive resources beyond the abilities of the patient group and thereby voids the increase in potential benefit therefrom. Furthermore, parents sometimes tend to both overestimate problems and downplay their child's condition in response to coping with the situation of having a child with pABI (58, 59). Therefore, both children and parents could experience an effect just by the fact that they are offered additional treatment and support in the chronic phase of the pABI (60). More precisely, our primary outcome measure was parental reports of EF in everyday life. Parents who participated in the interventions were blinded to the hypothesis, to begin with. Thus, the improvement detected with parental reports may reflect the parents' expectations or beliefs (61). As the blinding at the 2-year follow-up is still active, caregivers are not aware of which condition their children were assigned to. This may have contributed to the results in the sense that for the participants in the pGMT, some of the children may not have practised the skills and strategies acquired during the intervention (or have not been able to do so due to the cognitive demands) and that parental involvement through increased activity and engagement for the pBHW patient group might have offset the difference between the groups (61).

It is worth mentioning that the psychoeducative feature of pBHW further strengthened the original RCT. The active control was well-designed to match the pGMT in quality; thus, it is perhaps not surprising that participants in this group made reasonable progress both at the 6-month and 2-year follow-up. As we already have mentioned, we cannot know exactly why the pBHW group did as well as what they did in terms of sustained improvement. Similar to the pGMT, pBHW comprised many components that we do know are essential in operation for treatment to be effective, for instance, the involvement of family/caregivers throughout the process, the children/adolescents being assigned to smaller groups to make them feel safe, and tasks and content of the interventions being functionally oriented, potentially increasing the participants' motivation and commitment (62). The result is the features that are common for pBHW and pGMT rather than the components that distinguish the two interventions from one another could be the main explanatory factors to our findings. The effort in designing an equally effective intervention for the active control group can in fact impose a challenge (61). As already mentioned, self-awareness and insight into own condition are often reduced among individuals with ABI. However, those participants assigned to the active control group may have obtained essential knowledge and insight into their own condition that elevated their level of self-reflection. Education in such a sense was not given to the participants in the pGMT group, who had a different scope of their intervention. Potentially, the differences between the two interventions might have been levelled out due to this. As researchers before us have pointed out, for some of the participants in the study, the metacognitive demands set by pGMT might have surpassed the available cognitive resources (43). To achieve full comprehension or utilisation of the strategies learned in GMT, the individual needs self-awareness and reflective skills at a relatively high level (40), which are the same skills commonly found to be reduced in individuals with ABI. This could suggest that perhaps pGMT would have benefited from a general educative module in the initial stage of the intervention.

Along with internal factors typically associated with the subjects, external influences must be considered, such as the pGMT protocol. The adult GMT protocol has been found effective to improve EF and to improve quality of life in adult populations. However, the paediatric-adapted protocol is still in its infancy and potentially not optimally suited for use in paediatric populations yet. The age range in our study, the youngest children being 10 years of age and the oldest being 17 years, serves the pGMT protocol injustice. For the pGMT protocol to become more age-appropriate, it could very well need further adjustments.

A limitation of previous cognitive rehabilitation studies for children and adolescents with ABI has been that the majority have reported results at a group level and not individual success rates (9, 62, 63). Knowing the portion of participants that show clinically reliable change is of relevance to clinicians when recommending treatments. Hence, in the present study, reliable clinical change from the baseline (T1) to the 2-year follow-up (T4) was estimated (50). No differences were found between the treatment conditions in the number of participants that showed clinically reliable improvement after intervention. Interestingly, a few children demonstrated negative clinical change after both interventions. It is not likely the worsening in executive dysfunction in these subjects is a result of the interventions they were exposed to. It may not even be a worsening in symptoms, rather it could be an expression of the child becoming more aware of his/her condition. Whatever the cause, we believe it is important and ethical to report such negative findings.

Contrary to previous assumptions, that injuries to the immature brain were less detrimental than in adults due to plasticity, we now know that early development represents a particularly vulnerable period (24). It is suggested that the adjustments that happen in the affected and immature brains in terms of rewiring the remaining functioning neural networks could lead to a delay in disability (25). Many research studies indicate that injury severity, age at injury, and time since injury function are strong predictors of long-term outcomes across functional domains (2, 6, 7, 64–67). In recent years, fatigue is found to be a strong predictor alongside psychosocial and medical factors for the long-term outcome of children with ABI as well (18, 68, 69). Considering this, the variety and severity of symptoms following pABI and how they might contribute to a disability must be viewed within a biopsychosocial framework when trying to understand why some individuals worsen in the functioning level relative to what would be expected (10, 70).

### 4.1. Strengths and limitations

This is one of the only few studies investigating the long-term outcome of cognitive rehabilitation in terms of pGMT relative to a psychoeducative treatment in pABI. The present study represents an important contribution to the knowledge base for this clinical population given the robust RCT design and long-term follow-up. Our results should be interpreted with some caution, however. First, the high attrition rate in the last time point represents a threat to the validity of our study in terms of reduced statistical power and an increase in variance (71). However, our *post-hoc* analysis showed that the only difference between completers and non-responders was MI at the baseline. This difference was trivial, suggesting the chance of a selection bias to be minimal, although it should be kept in mind. Leading up to the 2-year follow-up (T4), we can only account for three of the participants that withdrew from further participation due to a worsening of their medical condition. Of the remaining participants, only one actively withdrew from the study. A strength of the study design, however, is the blinding and randomisation of participants to the respective treatment conditions and the high quality of treatment offered to the active control group. These methodological measures increase the likelihood of attrition, and missing data being attributed to random factors not known to us. As our *post-hoc* sensitivity analyses demonstrated, the models for the total sample of completers and non-responders—both with and without multiple imputations—pointed in the same direction. Some may question this method of analysis, but multiple imputations could be a good method to increase statistical power and less bias in the estimates than one would achieve by using merely a complete case analysis (53). Potentially, because of the COVID-19 pandemic and no physical or group meetings nor meetings with study therapists were offered to the participants leading up to the 2-year follow-up, this could be the reason for the high attrition rate. For future reference, efforts should be made between this and the upcoming 5-year follow-up trying to reintroduce participants back into the study.

Another limitation of our study that needs to be addressed is that we did not have a non-active control group, leaving us with no possibility to account for natural changes over time. The efforts of keeping non-specific factors as similar as possible (i.e., involvement of parents and teachers, professional attention, and group dynamics) may have masked the effects of pGMT. Prioritising having an active control group receiving an intervention designed to match the pGMT in quality, however, is in our opinion of greater importance from an ethical standpoint considering this clinical group to be especially vulnerable.

The choice of relying on self-reports and reports by proxy may be problematic as they may be biassed (e.g., overreporting/underreporting of symptoms, awareness, cognitive deficits, or social desirability bias) and potentially affect the accuracy and validity. A problem with research on children and EF has been the low construct validity of EF measures and/or tasks that have been applied (72). However, the BRIEF has proven to have good internal consistency and test–re-test reliability, and this questionnaire is more sensitive to dysexecutive problems compared to regular performance tests (73). Moreover, the BRIEF has been applied to and found useful in a wide range of clinical groups and settings, including children with TBI (16, 74). Although an inclusion criterion to the original RCT was having executive complaints in everyday life, the children in our study were within the normal range at the baseline in regard to the BRIEF scores (43). This could be the explanation as to why we only did find an overall improvement in our sample over time, and no difference between our intervention groups emerged.

Even though improvements can be observed in children following various interventions applying different delivery methods within the context of everyday routines of the child's life at home, there is strong support for the superiority of interventions operating within a family-supportive empowering framework with an emphasis on optimization of the family environment and provision of parenting strategies (35, 75). A strong side to this RCT was the involvement of parents and teachers throughout the intervention. In previous studies of adult populations, the intervention has usually been directed towards the individual participant. In our study, however, the intervention was not only intended for the children with ABI alone but their families as well and schools as represented by their teachers.

### 4.2. Future directions

Considering how most interventions are carried out through sequentially delivered individual or group sessions combined with homework in between sessions, new avenues of delivery need to be further explored. In recent years, more studies have explored the usefulness of online rehabilitation programs (76, 77) either as the sole treatment plan or combined with face-to-face, and the findings are intriguing. In addition to the delivery of the intervention, the content remains an important component to investigate more thoroughly. The interventions may vary in format depending on their theoretical framework, and therefore, the desired outcome measures will vary accordingly. Considering the effectiveness of GMT as an intervention to remediate EF in the context of pABI, an important question will be whether the duration of the initial intervention should be extended or distributed differently. The designated 14 h provided in the original RCT may have not been sufficient. Moreover, it would be reasonable to potentially incorporate a supplementary intervention plan for maintenance (78, 79). Alternative components that might be more suitable for the young subjects as well as alternative ways of delivering the intervention (e.g., use of external aids and cues that are especially designed towards young children and adolescents) should be considered.

Whether cognitive functioning is targeted as the level of function or on the level of activity will be an important issue to address (80). Interventions directed toward strengthening metacognitive skills like GMT will benefit from operating within a framework that focuses on real-life contexts as this will generate a potential generalizable effect on other areas of functioning that is important for life quality as well (63). However, being able to utilise metacognitive strategies in daily life requires certain essential skills that are not necessarily accessible to all individuals (38). Future studies should investigate more closely whether the cause is adverse effects of an intervention or alternative explanations. The combination of “natural progress” with both quantitative and qualitative alterations and the ability to compensate through the strengthening of alternative neural pathways could be the cause of the displayed functioning level rather than an intervention *per se*. This idea of plasticity is intriguing, granted not in its traditional format because plasticity in itself does not necessarily develop into new adaptive neural networks (24).

Time is a central factor considering children with ABI require long-term follow-up when addressing executive problems (16, 54), yet as we have experienced, long-term treatment and follow-up of this group is challenging. Children and adolescents are at a point in their lives with great physical, cognitive, and emotional change in which they seek to find their identity and place in the world. The added strain of brain injury will affect young people's sense of self, and with no surprise, will have a great influence on their engagement in rehabilitation and compliance (81). To accommodate for this, there is a potential of using a mixed methods design in future investigations. By combining the best of quantitative and qualitative research, it will be easier to ensure our research is patient-oriented, considering that the clinical utility of the interventions is the primary goal. Individual success rates including self-perceived function and quality of life are easily forgotten in the pursuit of significant large-scale studies.

## 5. Conclusion

Our findings align with that of adult populations, in that there is an improvement in EF following cognitive rehabilitation. Because an overall reduction in parent-reported executive dysfunction was found, but no significant difference in EF could be detected between the distinct treatment conditions in our study, for the time being, we cannot conclude that GMT in the context of pABI is superior to general psychoeducation. More research is therefore needed to establish whether pGMT leads to an additional improvement in EF for this clinical group.

## Data availability statement

The raw data supporting the conclusions of this article will be made available by the authors, without undue reservation.

## Ethics statement

Approval was obtained by the Regional Committee for Medical and Health Research Ethics (REC) Norway in 2017; reference 2017/772. Written informed consent was given by all participants before inclusion. Written informed consent to participate in this study was provided by the participants' legal guardian/next of kin. Written informed consent was obtained from the individual(s), and minor(s)' legal guardian/next of kin, for the publication of any potentially identifiable images or data included in this article.

## Author contributions

HS contributed to the analysis and interpretation of results, drafting of the manuscript, critical manuscript revision, and final approval. SA contributed to study design and manuscript editing. IH contributed to statistical analysis and manuscript editing. JS, RH, AB, TF, TR, and KR contributed to study design, data acquisition, and manuscript editing. JS was also the principal investigator and responsible for the funding of the present follow-up study. All the authors contributed to the article and approved the final version of the manuscript.
