# Supplementary material for: Cognitive rehabilitation in paediatric acquired brain injury—A 2-year follow-up of a randomised controlled trial
Source: Front Neurol. 2023 May 30;14:1173480. doi: 10.3389/fneur.2023.1173480 (PMC10267836; doi:10.3389/fneur.2023.1173480)
Supplement: Supplementary file 1 [file Table_1.docx]

| Supplementary BRIEF self-report | | | | | | | | |
| --- | --- | --- | --- | --- | --- | --- | --- | --- |
| Measure  *Mean* [95%CI] |  | Baseline  T1 | 8-week  T2 | 6-month  T3 | 24-month  T4 | Group  *p* | Time  *p* | Group *time  *p* |
| BRIEF-BRI | GMT  BHW  Total | 57.1 [51.8-62.4]  58.2 [52.4-64.1]  57.7 [53.7-61.6] | 55.0 [48.4-61.6]  57.7 [50.3-65.0]  56.4 [51.4-61.3] | 55.7 [49.2-62.1]  55.5 [48.3-62-7]  55.6 [50.7-60.4] | 59.4 [52.2-66.6]  55.0 [46.7-63.3]  57.2 [51.7-62.7] | .961 | .663 | .576 |
| BRIEF-MI | GMT  BHW  Total | 69.5 [64.2-74.8]  71.0 [65.1-76.9]  70.3 [66.3-74.3] | 66.8 [60.0-73.7]  68.8 [61.3-76.3]  67.8 [62.7-72.9] | 66.5 [60.2-72.9]  68.1 [61.0-75.2]  67.3 [62.6-72.1] | 66.7 [59.4-73.9]  67.1 [58.7-75.5]  66.9 [61.4-72.5] | .731 | .273 | .994 |

2-years outcome for T4-sample: Fixed effects estimates, and estimated means with 95% CI for pGMT, pBHW, and total T4 sample.
